# Repeatability of metabolic tumor burden and lesion glycolysis between clinical readers

**DOI:** 10.3389/fimmu.2023.994520

**Published:** 2023-02-15

**Authors:** Jung W. Choi, Erin A. Dean, Hong Lu, Zachary Thompson, Jin Qi, Gabe Krivenko, Michael D. Jain, Frederick L. Locke, Yoganand Balagurunathan

**Affiliations:** ^1^ Department of Diagnostic Imaging and Interventional Radiology, H Lee Moffitt Cancer Center, Tampa, FL, United States; ^2^ Blood and Marrow Transplant and Cellular Immunotherapy, H. Lee. Moffitt Cancer Center, Tampa, FL, United States; ^3^ Division of Hematology and Oncology, University of Florida, Gainesville, FL, , United States; ^4^ Cancer Physiology, H. Lee. Moffitt Cancer Center, Tampa, FL, United States; ^5^ Tianjin Medical University Cancer Institute and Hospital, Tianjin, China; ^6^ Biostatistics & Bioinformatics, H. Lee. Moffitt Cancer Center, Tampa, FL, United States; ^7^ Machine Learning, H. Lee. Moffitt Cancer Center, Tampa, FL, United States

**Keywords:** metaboloic tumor burden, CART-therapy, lymphoma – diagnosis, imaging in CAR-T therapy, reproducible imaging biomarkers

## Abstract

The Metabolic Tumor Volume (MTV) and Tumor Lesion Glycolysis (TLG) has been shown to be independent prognostic predictors for clinical outcome in Diffuse Large B-cell Lymphoma (DLBCL). However, definitions of these measurements have not been standardized, leading to many sources of variation, operator evaluation continues to be one major source. In this study, we propose a reader reproducibility study to evaluate computation of TMV (& TLG) metrics based on differences in lesion delineation. In the first approach, reader manually corrected regional boundaries after automated detection performed across the lesions in a body scan (Reader M using a manual process, or manual). The other reader used a semi-automated method of lesion identification, without any boundary modification (Reader A using a semi- automated process, or auto). Parameters for active lesion were kept the same, derived from standard uptake values (SUVs) over a 41% threshold. We systematically contrasted MTV & TLG differences between expert readers (Reader M & A). We find that MTVs computed by Readers M and A were both concordant between them (concordant correlation coefficient of 0.96) and independently prognostic with a P-value of 0.0001 and 0.0002 respectively for overall survival after treatment. Additionally, we find TLG for these reader approaches showed concordance (CCC of 0.96) and was prognostic for over -all survival (p ≤ 0.0001 for both). In conclusion, the semi-automated approach (Reader A) provides acceptable quantification & prognosis of tumor burden (MTV) and TLG in comparison to expert reader assisted measurement (Reader M) on PET/CT scans.

## 1 Introduction

Diffuse large B-cell Lymphoma (DLBCL) is the most frequently observed lymphoma subtype and accounts for about 40% of new cases of lymphoma. This aggressive form of lymphoma spreads systemically involving organs other than lymph nodes with a 5-year survival of about 64% for all patients that drops to 57% with distant metastases and further with risk factors such as age, lactate dehydrogenase (LDH) levels, disease stage, and patients’ performance status (1–3). Disease management of DLBCL can be a challenge due to heterogeneous disease characteristics and a poor prognosis when first line treatment fails. Advancements in Positive Emission Tomography (PET) imaging using ^18^F fluorodeoxyglucose (FDG) in lymphoma have allowed better disease staging, characterization (4, 5) and response assessment (6). Recent evolutions in the field have allowed adoption of PET combined with computed tomography (PET/CT) to become a standard for disease assessment (7, 8). There have been several studies that have shown utility in using PET/CT for response assessment in lymphoma (9–12). A clinical assessment of response includes a five-point scale (5-PS) to assess degree of response at mid to end treatment stages, based on qualitative experts evaluation of imaging scans (13). This was first recommended as a reporting criteria in the first PET workshop on Lymphoma in Deauville, France in 2009, and has been adopted in many clinical trials (14, 15). The continually improving resolution of imaging data has allowed development of alternative response measurement criteria (16). It has been well documented that interobserver biases in most widely used radiological lesion measurement, Response Evaluation Criteria in Solid Tumors (RECIST) criteria are influenced by several factors, such as scan quality, image resolution, training, and other minor factors (17, 18). In FDG/PET imaging, the standardized uptake value (SUV) has been successfully used as a measure of metabolic activity for disease diagnosis and therapy assessment, but suffers from inter-patient variability, intra-tumoral variability, and procedural related factors, which has led to a debate on the extent of its clinical usage (19). Recent developments of high-resolution imaging have allowed for the development of Metabolic Tumor Volume (MTV) and Total Lesion Glycolysis (TLG) as potential exploratory parameters for characterization that provides information about the 3D structure of the tumoral, tumor viability and its spatial variations (20–22). These metrics have been independently shown to be prognostic for treatment response in lymphoma after chemotherapy (23, 24). Recently, we demonstrated that MTV (on baseline patients) is prognostic for DLBCL treatment response to axicabtagene ciloleucel (axi-cel), a CD19 targeted Chimeric Antigen Receptor T cell (CAR-T) therapy when used as a third or later line of therapy (25). The MTV allows ensemble estimates of active tumor volume regions and its microenvironment. While the TLG provides an assessment of metabolic activity of the tumor, a valuable metric to assess gross level of active lesions, which is defined as a product of MTV and an average of standard uptake value (SUV_max_), a fixed value threshold to identify metabolic tumor regions is still considered reliable (26). These metrics have not been standardized and the computation is influenced by several factors that include methods for delineation, assessing metabolic levels that has been well shown by many studies to evaluate reader agreements and variability, essential for clinical adoption (26–29).

In this study we propose to study the reader repeatability of MTV and TLG assessment with variations in approaches (Reader- A & M), in which one reader manually corrects tumor boundaries (Reader M), while the other reader would use semi-automated methods (Reader A). We contrast both approaches at various size ranges and assess the prognosis of patients after treatment of CAR-T immunotherapy. In this study we show use of semi-automated methods would allow faster evaluation of these metrics, essential for clinical adoption.

## 2 Material and methods

### 2.1 Patient cohort

The retrospective review of patient records was approved by the University of South Florida’s Institutional Review Board (IRB) for the research study. We accessed ninety-six Large B Cell Lymphoma (LBCL) patients with relapsed or refractory disease who received axi-cel treatment from May 2015 to June 2019, treated with the CAR T cell therapy, axicabtagene ciloleucel (axi-cel). Clinical outcomes based upon PET/CT scan derived MTV for the same cohort of patients was previously reported (25). The clinical records and imaging data (^18^F-FDG PET/CT scans) for the patients were obtained for the study. We assessed the baseline scans of these patients prior to the start of treatment. We abstracted patient records to obtain survival and vitals data. We evaluated the outcome variables as overall survival and progression free survival after treatment at 1 year. Some patients received bridging therapy during the manufacture of their CAR-T cells (process takes approximately 3 weeks), which we defined as any lymphoma specific therapy given after apheresis but prior to the start of fludarabine cyclophosphamide chemotherapy for lymphodepletion before CAR T-cell infusion. In our study about half our patients received bridge therapy (n=46). Details on our patient cohort used for the study is described in **Table 1**.

**Table 1:** Patient cohorts clinical characteristics at baseline.

Characteristics	N=96
Age (mean, median, std.)	60.5 Years (64, 12.4)
Sex (male/female)	61/35
**LDH** (before conditioning, day -6 or close) (mean, median, std.)	393.2 (275.5, 322.04)
ECOG	
0-1	78
2-3	18
Stage	
I/II	22
III/IV	74
Bridging Therapy	
Yes	46
Chemotherapy/targeted therapy	12
Steroids	9
Radiation	5
Chemotherapy/targeted therapy ± steroids ± radiation	20
Received prior to CT/PET	24
Axi-cel Administration	
Trial (cancer center) Commercial/Consortium (sponsored)	22 76

### 2.2 Metabolic tumor burden and total lesion glycolysis

Patients baseline PET/CT whole body scans performed prior to axi-cel treatment were obtained to compute Metabolic Tumor Burden (MTV) and Total Lesions Glycolysis (TLG). We used custom tools implemented on MIM PACS (version 6.8.4, MIM Software, Cleveland, OH) to identify and compute the metrics. The custom workflow was used to identify abnormal regions in the scan with FDG metabolic activity over a predetermined level within an user identified volume in normal liver (mean volume around 13.9 ml). In our workflow, a reference region in the normal liver was located by a human expert using a single click. A fixed radius (3cm sphere) was placed at the selected liver region (single click) was used to estimate the baseline mean, with a detection threshold cutoff of 2 standard deviations over the mean liver as recommended by ^18^FDG-PET detection criteria (30–33) was used to identify abnormal regions.

The lesions were verified by the expert readers in the following ways; A) Reader -M, a medical oncology fellow (6 years of clinical experience), alter the regional boundaries based on uptake revealed on PET image. In some cases, Reader - M had the option to add new lesions that were not identified by threshold- based detection. Metrics measured using this regional boundary will be referred to as Reader M (or *manual*). The lesion boundary edits, any additional marking were overread by a research radiologist (HL or JQ) to be consistent with the clinical workflow. B) radiologists (JC & HL, JQ over 16 years, 10 years and 7 years of clinical experience, respectively), chose to accept or remove false detected lesions after automated detection, but did not alter the lesion boundaries, or add even if the detection method does not find the lesion boundary. Metrics measured using this regional boundary will be referred to as Reader – A (or *auto*).

**Figure 1:**
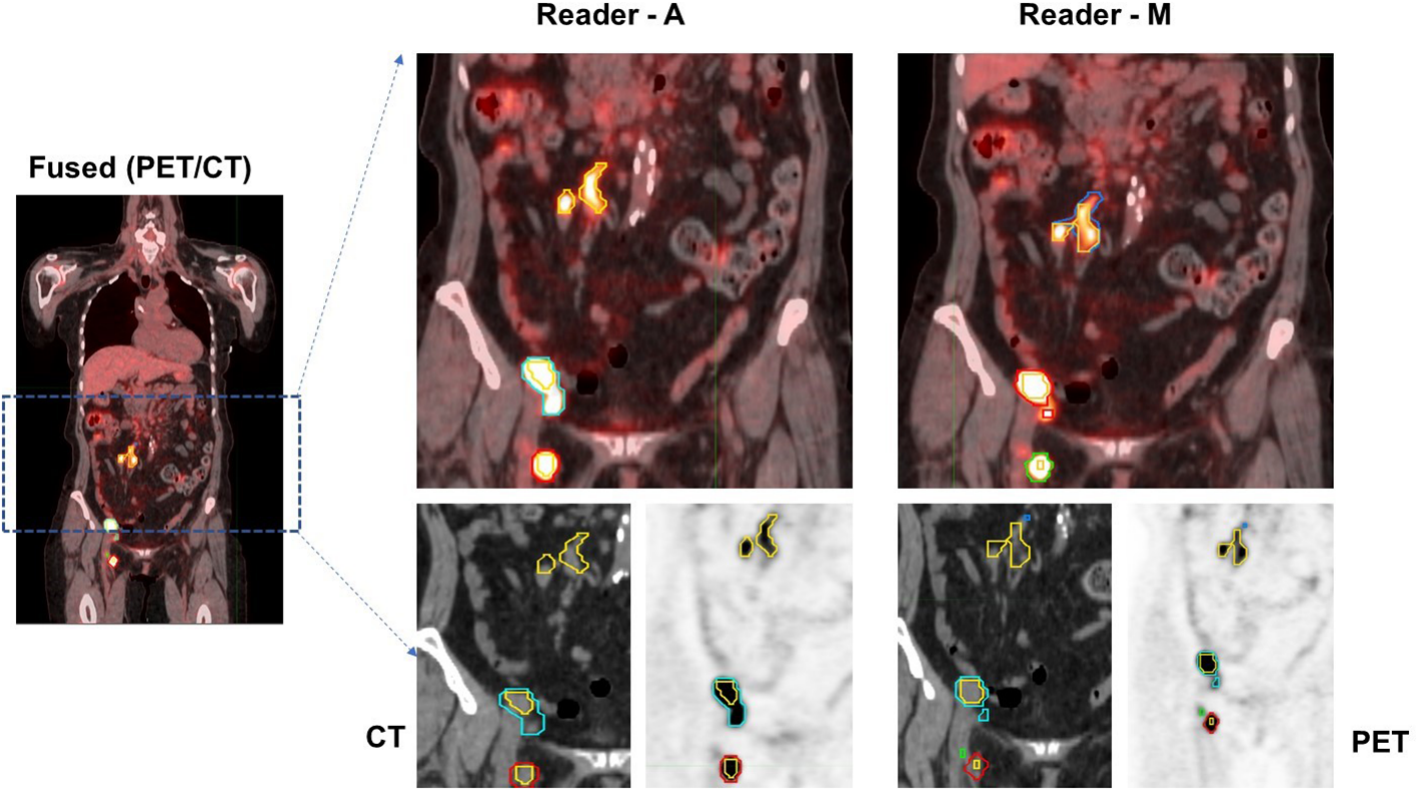
Patients image scan (PET/CT) with lesion delineation shown on a representative slice. Multiple boundaries for a lesion indicate original lesion segmentation (reader M and reader A) and corresponding 41% cut-point (inner boundary for a lesion). Lesions with overlapping regions (original and 41% cut-point) will show one boundary. In the example case, using Reader M’s approach, MTV was estimated to be 28.07 ml (TLG: 328.93 SUV*ml) while Reader A’s approach estimated MTV was 23.25 ml (TLG: 315.25 SUV*ml), with a difference of 17.2% in their MTV estimates.

In both approaches, physiologic uptake (false detections) were removed that could be in metabolically active, non-malignant organ sites (bladder, brain etc.) or other nonmalignant processes (e.g. degenerative disk disease or muscle activity). The readers assessed these lesions independently. The readers had access to clinical reports at the time of assessment but not to the lesions or its boundaries marked by the other reader. **Figure 1** shows an example case with the readers’ assessment.

The final lesion boundary was converged by combining voxels that are over the 41% of SUV_max_ at the individual lesion level. The cumulative sum of voxels across the lesions (over 41% SUV max) in the body scan are reported as Metabolic Tumor Volume. Total Lesion Glycolysis is defined as a product of MTV and an average SUVmax, which provides a measure of gross extent of tumor metabolic activity. We used workflows implemented in MIM (version 6.7, MIM software Inc) for MTV ad TLG computations. The expert evaluation would follow prior to TMV computation. The workflow tools are specific to MIM PACS and will be shared through the vendor.

### 2.3 Discordance in lesion identification

Identifying lesions using CT and PET scans have been shown to improve lesion detection in many clinical studies including lymphoma (34–36). The functional aspect of FDG-PET based imaging provides metabolic information of tumor regions used by the oncologist (37). There are many instances where lesions can be missed or wrongly assessed as an active lesion. We list below potential major reasons for discrepancy between clinical reader-based approaches (i.e., Reader M & A).

a. *Metabolically active regions from normal physiology*: It has been well documented that 18F-FDG is well absorbed by metabolically active organs that show up as tracer avid regions on PET images, such as the brain, heart, and bowel (37). The MIMS automated region selection algorithm does not discriminate between metabolically active organs and tumor, therefore in its current state it cannot be completely automated.
b. *Inflammation related regions*. For regions that have metabolic activity on imaging, one must exclude the possibility that the activity is secondary to a benign inflammatory process, as issues with accurate discrimination have been well acknowledged (38). A clinical radiologist may look for patterns of FDG uptake on the PET or fused PET/CT images, with comparison to tissue density and anatomy on the CT images.

### 2.4 Statistical analysis

The MTV and TLG metrics on the patient scan were computed following selections made by the two readers, with manual edits used by Reader M previously described (25) and semi-automated methods followed by Reader A. We compared the concordance between the approaches using concordance correlation coefficient which measures the deviation from the ideal mid-point (45-degrees line) between the value of the estimates, across the population (39). We used Altman-Bland’s plots to investigate the difference across the population (40). This approach elegantly compares two variables for agreements to understand the bias between the measurements by comparing the measurement means to the difference. The method provides analytical estimates to create confidence limits on the agreement or disagreements and allows identification of individual samples with these bounds. The analysis was repeated for various categories to identify confounders such as number of lesions in a patient scan between two readers.

Clinical outcomes for these patients after axi-cel CAR-T treatment was collected that include follow up scans and patients’ survival (vital status), performance status. The overall survival (OS) and progression free survival (PFS) were computed based on the time between axi-cel infusion until death or disease progression, or the last date the patient was contacted or known to be alive. Kaplan-Meier (KM) survival plots were drawn by dividing the population using MTV (or TLG) into two groups based on median value split for these individual estimates (high or low values of MTV or TLG). The significance of these cohort populations was assessed based on a log-rank statistical test. Hazard ratios (HRs) and 95% confidence intervals (CI) were reported. Cox regression analysis was performed for these variable estimates independently for manual and auto estimates of MTV & TLG to assess the risk factors of these assessments to survival time (OS) or progression (PFS). The KM plots and Cox regression models were repeated for these variable estimates using auto and manual approaches. The overall response rates including partial response (PR) and complete response (CR) to therapy were reported. The incidence of maximum cytokine release syndrome (CRS) was abstracted from the patients clinical records that use the standard assessment criteria (41). All the statistical analysis was performed using R studio packages (42).

## 3 Results

**Figure 2:**
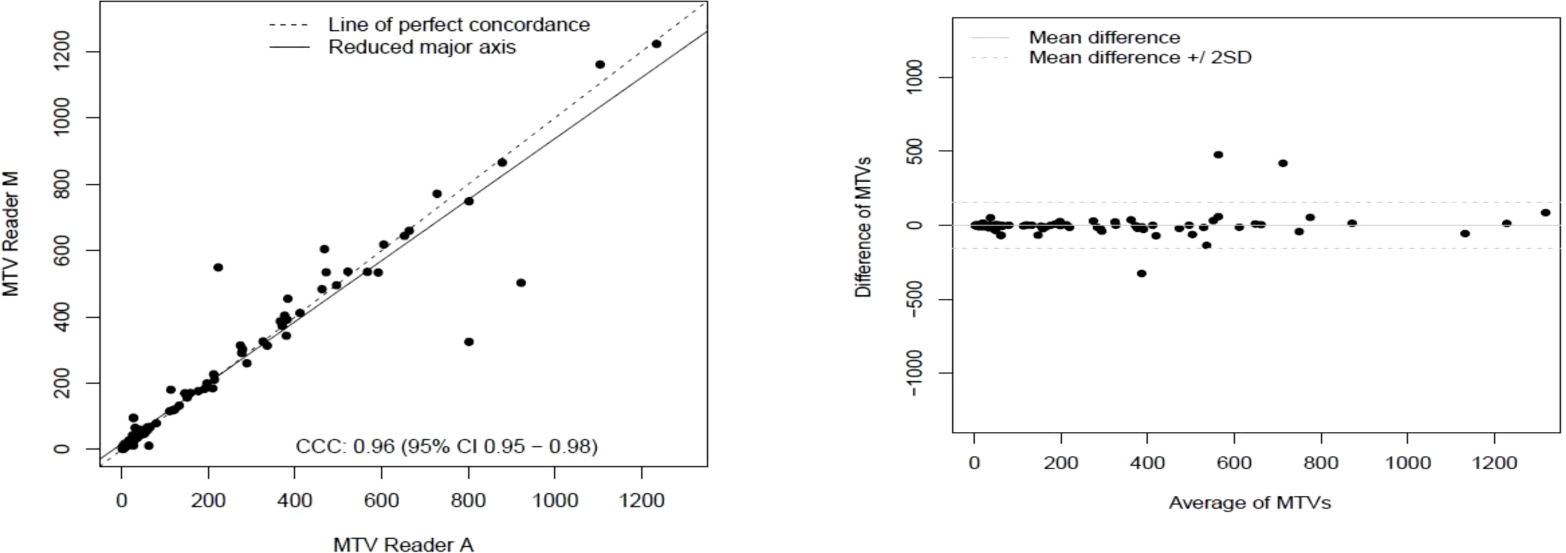
Comparison of estimates of metabolic tumor volume (MTV) between two readers (Reader A, Reader M) shown using a) scatter plots b) difference plots.

**Figure 3:**
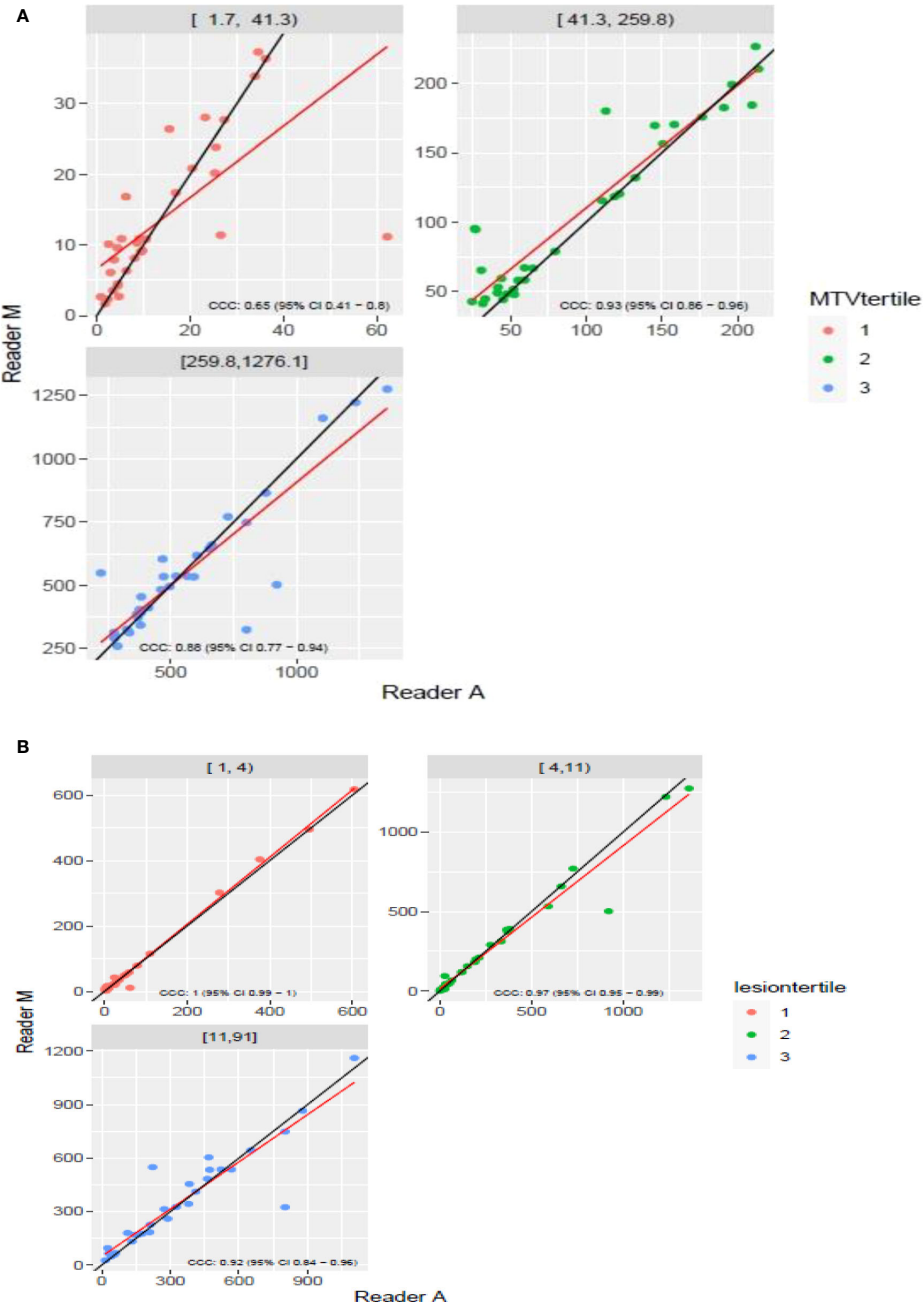
Comparison of estimates of metabolic tumor volume (MTV) between two readers (Reader A, Reader M) using scatter plots for three sub ranges obtained using(a) [**(A)** diving Reader M’s estimates **(B)**] number of lesions.

**Figure 4:**
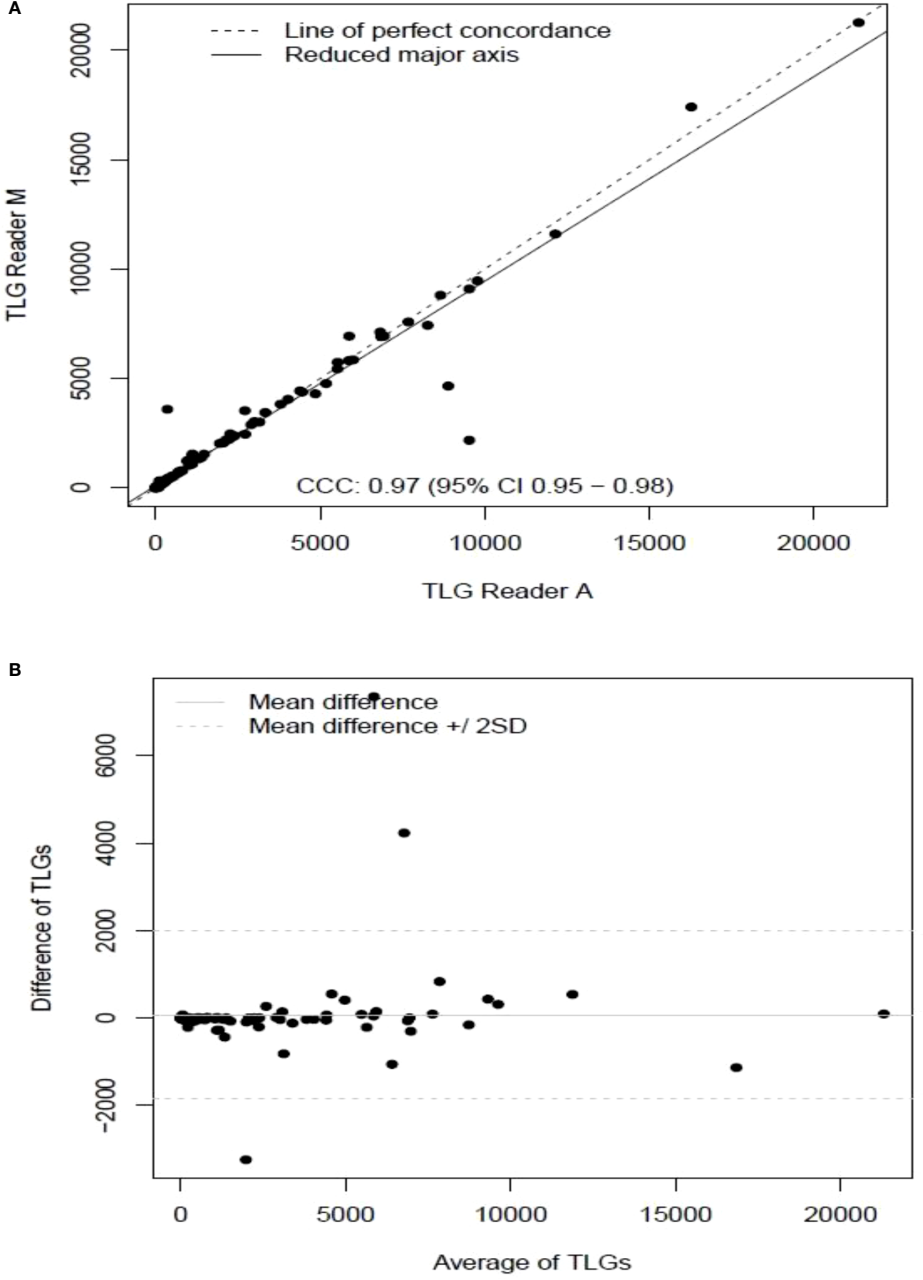
Comparison of estimates of tumor lesion glycolysis (TLG) between two readers (Reader A, Reader M) shown using **(A)** scatter plots **(B)** difference plots.

**Figure 5:**
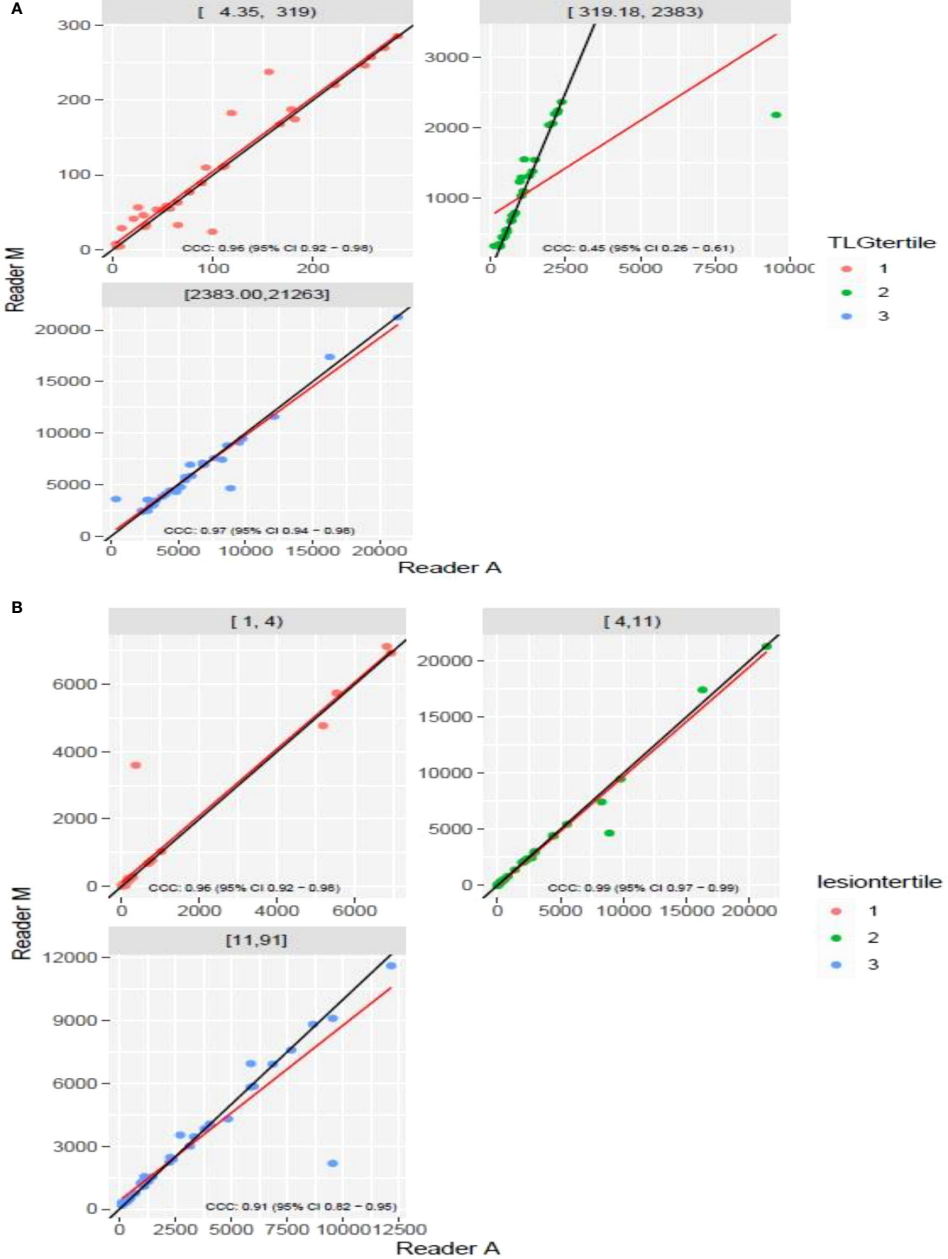
Comparison of estimates of tumor lesion glycolysis (TLG) between two readers (Reader M, Reader A) using scatter plots for three sub ranges obtained using(a) **(A)** dividing Reader M’s estimates **(B)** number of lesions.

**Figure 6:**
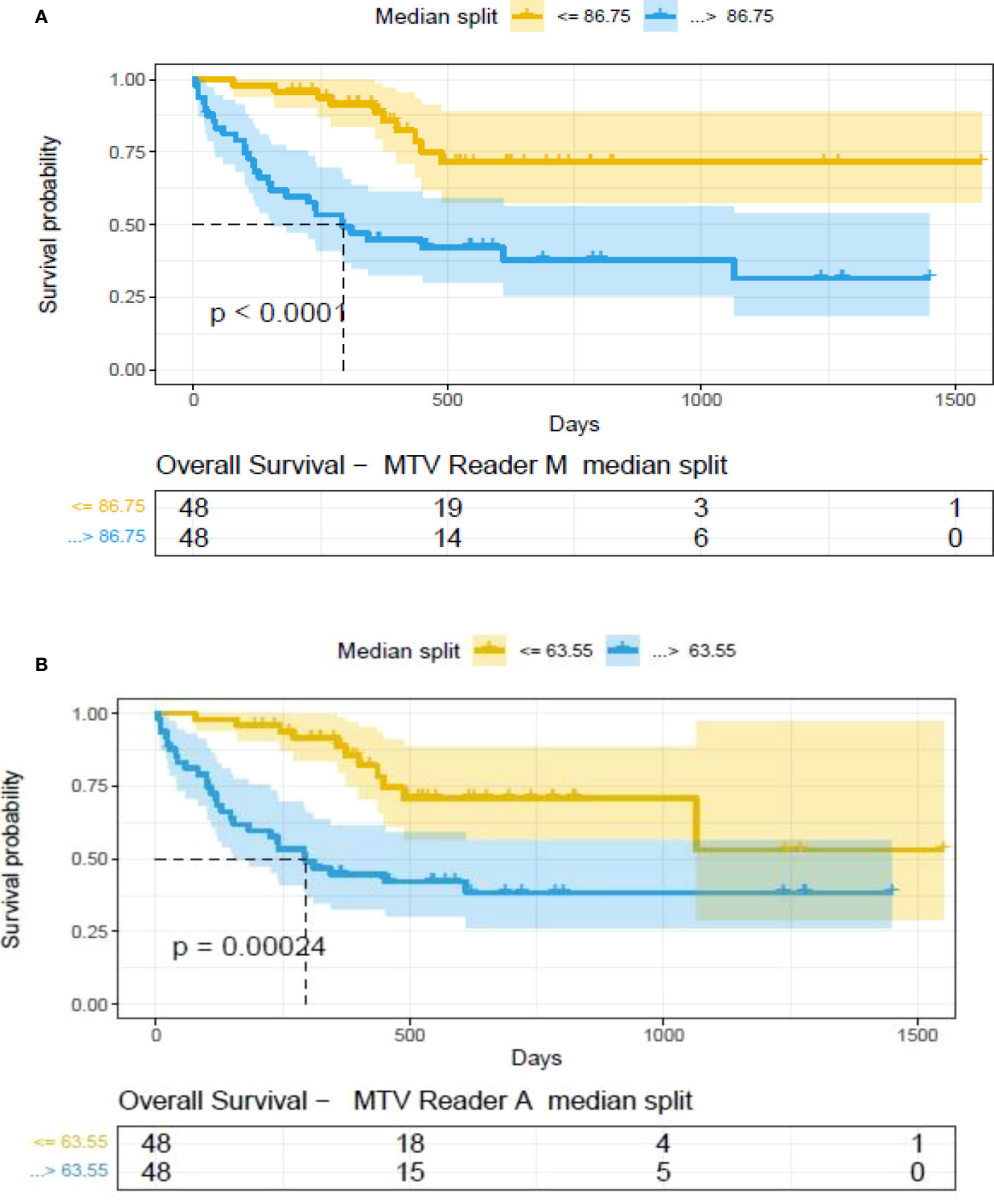
Survival difference between patient groups obtained using median split of Metabolic Tumor Volume (MTV) estimates obtained by **(A)** Reader -M (manual) and **(B)** Reader -A (auto), shown using Kaplan Meier plots. Significance between the patient groups computed using log-rank testing.

**Figure 7:**
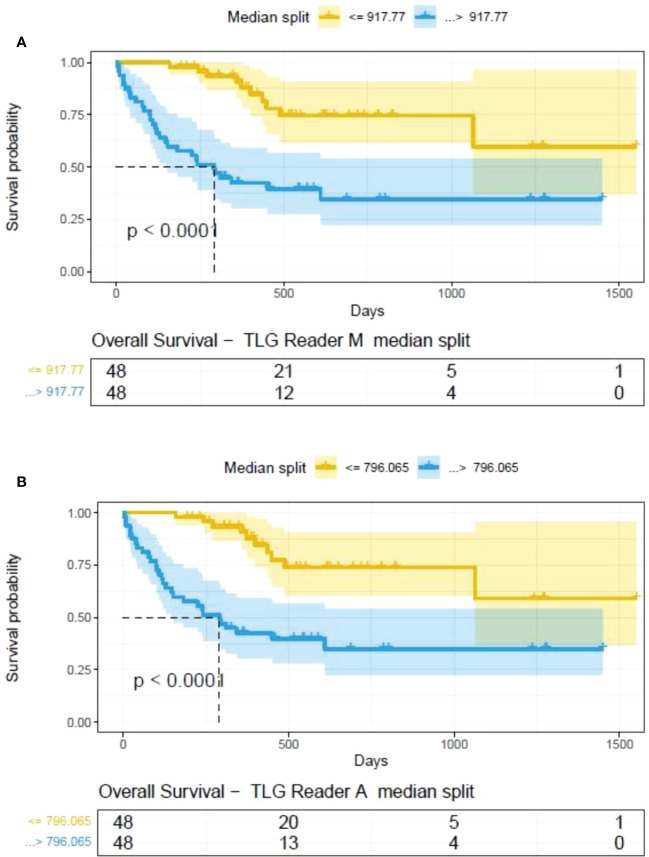
Survival difference between patient groups obtained using median split of Tumor Lesions Glycolysis (TLG) estimates obtained by **(A)** Reader -M (manual) and **(B)** Reader -A (auto), shown using Kaplan Meier plots. Significance between the patient groups estimated using log-rank statistical test.

**Figure 8:**
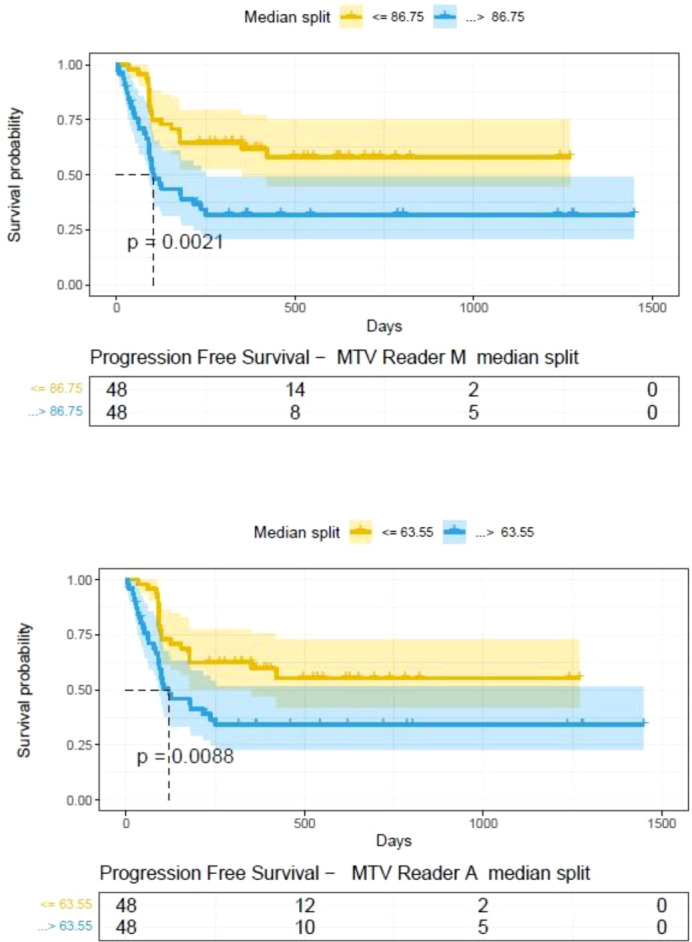
Progression free survival between patient groups obtained using median split of Metabolic Tumor Volume (MTV) estimates obtained by **(A)** Reader -A (auto) and **(B)** Reader -M (manual), shown using Kaplan Meier plots. Significance between the patient groups computed using log-rank testing.

**Figure 9:**
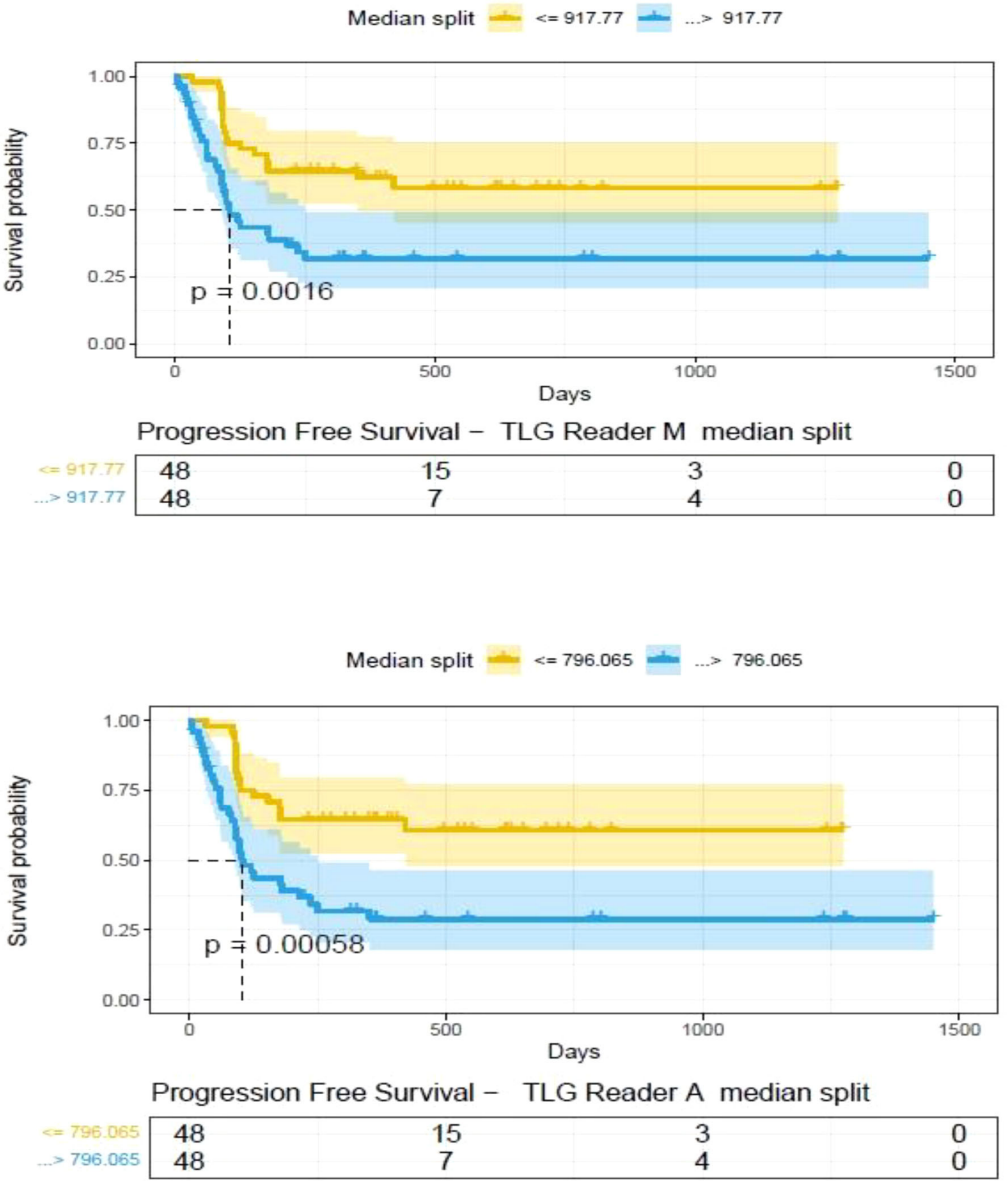
Progression free survival between patient groups obtained using median split of Tumor Lesion Glycolysis (TLG) estimates obtained by **(A)** Reader -A (auto) and **(B)** Reader -M (manual), shown using Kaplan Meier plots. Significance between the patient groups computed using log-rank testing.

**Figure 10:**
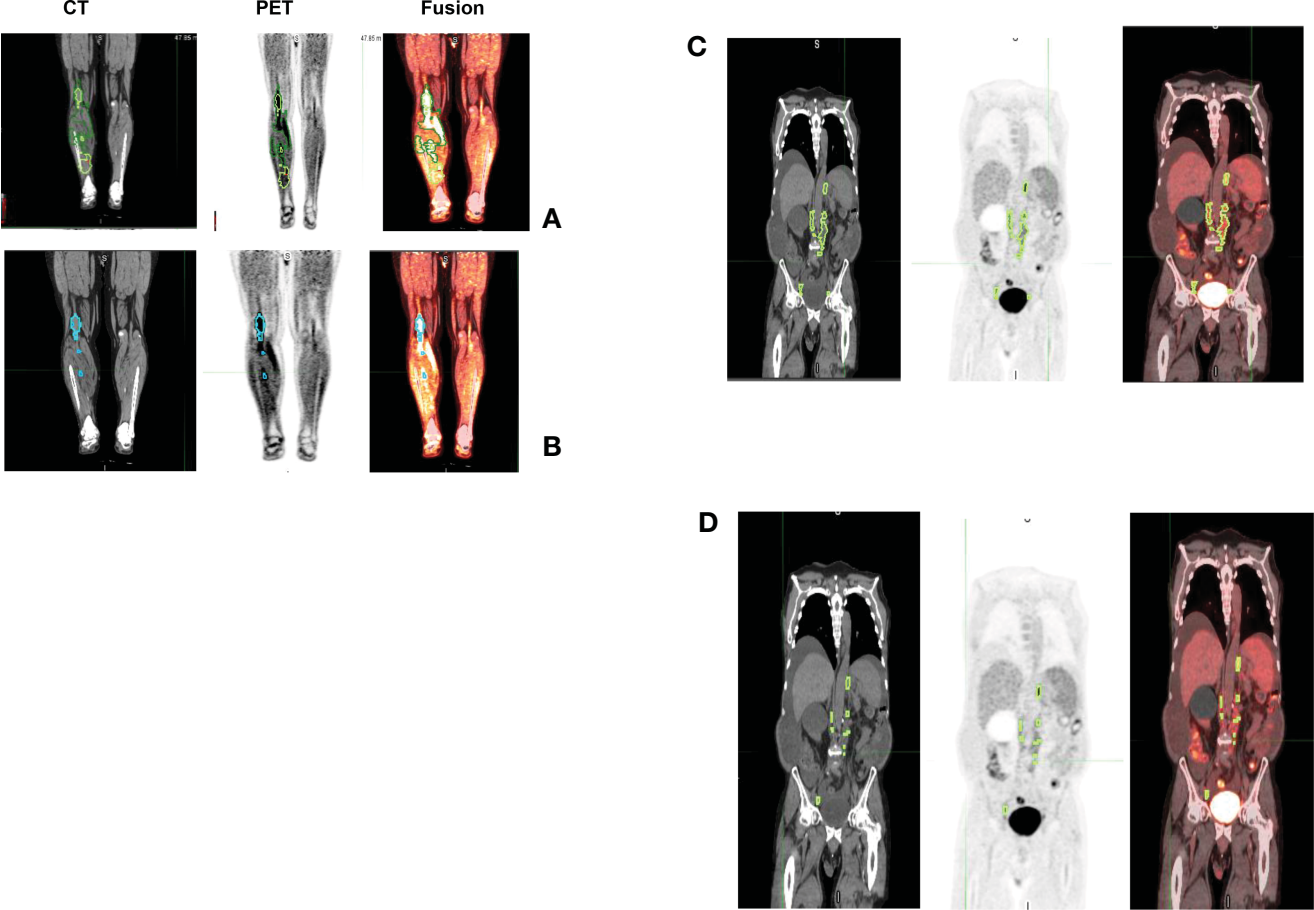
Representative patient scans (CT, PET, Fusion) with lesions detected using manual and auto procedure that showed larger differences in metabolic tumor burden. Estimated MTV using manual (case 1) was 94.62ml (TLG 352.68) (see **A**). Using auto (case 1) we estimated it to be 27.09ml (TLG 272.52) (see **B**). While, in case 2 we estimated MTV using manual (case 2) to be 95.37 ml (TLG 327.59) (see **C**) and using auto we estimated it to be 20.3ml (TLG 116.27) (see **D**).

The clinical characteristics of the cohort used in the study are described in **Table 1**. About 47% of the study patients received bridging therapy after apheresis to collect cells for manufacture of axi-cel, but prior to start of lymphodepletion given prior to axi-cel. We obtained the baseline PET/CT scans and MTV and TLG were computed using two approaches (manual and auto). The MTV estimates showed a concordance correlation coefficient (CCC) of 0.963 between the estimates. Bland-Altman’s plot shows outliers (see **Figure 2**). We further divided the MTV metric into tertiles based on lesion volume (in ml), grouped into [1.7, < 41.3), [41.3 <259.8), [259.8, <1276.1) and the concordance between the Readers (M and A) for the groups were estimated to be 0.649, 0.927 and 0.882 for the respective groups. The cohort was further divided into tertiles based on the number of lesions (in each group, manual & auto). The group range was [≥1, < 4), [≥4, <11), [≥11, 91], and the concordance correlation between the Readers (1 & 2 or M & A) for the groups were 0.997, 0.974 and 0.92 (see **Table 2a**). The scatter plots show a visual comparison of the estimates between the readers (1 & 2 or M & A) and concordance lines (in black) with regression fit (in red) for these sub cohorts (**Figure 3** **).** The TLG metric showed a concordance of 0.965 between the readers (1 & 2 or A and M), see **Figure 4**. The tertile on TLG metrics had a range of [≥4.35, < 319), [≥319, < 2383), [≥2383, <21263), and these groups showed a concordance of 0.96, 0.45 and 0.97 respectively. When the cohort was divided into tertiles based on number of lesions, the ranges were [≥1, <4), [≥4, <11], [≥11, <91), the concordance between the readers (M & A) for these range were 0.96, 0.97 and 0.91 respectively, (see **Table 2b**). We compare the TLG estimates between concordance line (in black) with regression fit (in red) for these sub cohorts (**Figure 5** **).** A cox regression model was developed using MTV (univariate) & multivariable MTV, LDH and ECOG status in immunotherapy patients to assess risk to over-all survival after CART treatment. We find MTV shows increased hazard risk to over-all survival at 0.14% using Reader M’s estimate and 0.10% using Reader A’s estimate (for Univariate model). While the TLG -Reader M showed a risk of 0.008% and TLG-manual had a risk of 0.007% for over-all survival (see **Table 3** and **Supplementary Tables 1**, **2**). In this study, we used the median value of MTV estimates to divide the cohort and assessed their survival prognosis, the significance estimated using a log-rank test. Using MTV –(reader M) the p value was 0.0001 and using MTV –(reader A) the p-value was 0.0002 (see **Figure 6**). The KM plot cut point for reader 1 using MTV (reader M) was 86.75 mL, while reader using MTV (reader A) methods was 63.55 mL. The MTV metric showed significant prognostic difference for progression free survival, 1 year after CAR-T, as p-values were 0.0021 and 0.0088 for Reader M and A, respectively. The TLG estimate was used to divide the cohort based on median estimate and assess the significance using log-rank test. We estimated a p- value of 0.0001 for both TLG-Auto and TLG-manual (see **Figure 7**). MTV and TLG's significant prognosis for progression free survival (PFS) are shown in **Figures 8**, **9** respectively. The TLG metric showed significant prognosis for progression free survival (1 year after CAR T) with an estimated p-value using log rank test of 0.0016 and 0.00058 for readers M and A respectively. There were seven patients that showed difference in prognosis comparing inference between MTV (M & A) and TLG (M&A) in our cohort (see **Table 4**), with corresponding real patient PET/CT scan shown as an example (see **Figure 10**). Comparing the MTV (M &A) there were four patients switched between prognosis groups (see **Table 4a**). While comparing TLG (M & A), one patient switched prognosis groups (see **Table 4b**). While comparing MTV & TLG (both manual) there were five patients that switched prognosis grouping (see **Table 4c**). In MTV & TLG (both auto) there were four patients that switched prognosis (see **Table 4d**).

**Table 2:** Concordance between two approaches (manual and auto) for a) tumor metabolic burden (MTV) and b) total lesion glycolysis (TLG).

(a) MTV estimate concordance between Reader A & M		
	All data	0.96 [0.946, 0.975]
#	Tertile across MTV (M)	Rho (C est)
	[1.7, 41.3)	0.649 [0.414, 0.802]
	[41.3, 259.8)	0.923 [0.859, 0.963]
	[259.8,1276.1)	0.882 [0.774, 0.939]
MTV estimates across number of lesions		
#	Lesion count	Rho (C est)
	[1, 4)	0.9988 [0.993, 0.998]
	[4, 11)	0.974[0.949, 0.987]
	[11,91)	0.919[0.843, 0.959]
(b) TLG estimate concordance between Reader A & M		
	All data	0.965 [0.949, 0.977]
#	Tertile across TLG (M)	Rho (C est)
	[4.35, 319)	0.958 [0.916, 0.978]
	[319.2, 2383)	0.453[0.263, 0.609]
	[2383, 21263]	0.969[0.938, 0.984]
TLG estimates across number of lesions		
#	Lesion count	Rho (C est)
	[1, 4)	0.959 [0.918, 0.980]
	[4, 11)	0.974 [0.946, 0.987]
	[11,91)	0.908 [0.822, 0.953]

**Table 3:** Cox regression model to assess hazard risk of MTV and TLG metrics to patient’s overall survival (OS) (*see a & b*) and progression free survival (PFS) (*see c & d*).

(a) Univariate Cox Model (OS) – MTV		
Variable	Hazard Ratio	P-value
MTV (Reader M)	1.00147	0.000356
*Multivariable*		
MTV (Reader M)	1.00125	0.01
LDH	1.00026	0.603
ECOG	1.483	0.104

MTV (Reader A)	1.00111	0.00656
*Multivariable*		
MTV (Reader A)	1.00088	0.089
LDH	1.0003	0.511
ECOG	1.568	0.057
(b) Univariate Cox Model (OS) – TLG		
Variable	Hazard Ratio	P-value
TLG (Reader M)	1.00008514	0.00857
*Multivariable*		
TLG (Reader M)	1.0057	0.156
LDH	1.00048	0.306
ECOG	1.53	0.073

TLG (Reader A)	1.0000734	0.0313
*Multivariable*		
TLG (Reader A)	1.0041	0.888
LDH	1.00049	0.328
ECOG	1.579	0.050
(c) Cox Model (PFS) – MTV		
Variable	Hazard Ratio	P-value
MTV (Reader M)	1.0013	0.0014
*Multivariable*		
MTV (Reader M)	1.00145	0.00348
LDH	0.9997	0.701
ECOG	0.9337	0.777
		
MTV (Reader A)	1.00107	0.00773
*Multivariable*		
MTV (Reader A)	1.0012	0.0152
LDH	0.999	0.627
ECOG	0.978	0.926
(d) Cox Model (PFS) – TLG		
Variable	Hazard Ratio	P-value
TLG (Reader M)	1.0071	0.027
*Multivariable*		
TLG (Reader M)	1.0069	0.0808
LDH	1.00004	0.9347
ECOG	0.985	0.952
		
TLG (Reader A)	1.0066	0.0438
*Multivariable*		
TLG (Reader A)	1.007	0.11
LDH	0.9998	0.857
ECOG	1.0052	0.983

**Table 4:** Patient level discrepancy in prognostic decisions.

a. MTV Prognosticator		
Patient#	MTV (M) (cut pt. 86.75)	MTV (A)(cut pt. 63.55)
**1**	Poor (94.62)	Better (27.09)
**4**	Better (66.74)	Poor (64.84)
**6**	Poor (95.37)	Better (26.3)
**7**	Better (78.88)	Poor (79.51)
b. TLG Prognosticator		
Patient#	TLG (M)(cut pt.917.77)	TLG (A)(cut pt.796.06)
**3**	Poor (3598)	Better (360.15)
c. Compare MTV & TLG based prognosticator (Reader M )		
Patient#	MTV (M)(cut pt.86.75)	TLG (M)(cut pt.917.77)
**1**	Poor (94.62)	Better (352.68)
**2**	Poor (169.56)	Better (755.96)
**3**	Better (33.91)	Poor (3598)
**5**	Better (59.39)	Poor (1236.68)
**6**	Poor (95.37)	Better (327.59)
d. Compare MTV & TLG based prognosticator (Reader A)		
Patient#	MTV (A)(cut pt. 63.55)	TLG (A)(cut pt.796.06)
**2**	Poor (145.33)	Better (715.48)
**4**	Poor (64.84)	Better (461.37)
**5**	Better (59.39)	Poor (958.6)
**7**	Poor (78.88)	Better (692.98)

Comparing decisions with: a) MTV based prognosticator independently applied using reader-M and reader-A’s estimates, b) TLG based prognosticator independently applied using reader M and reader A’s estimates, c) compare MTV and TLG based prognosticator with reader M’s estimate d) compare MTV and TLG based prognosticator with reader A’s estimate.

## 4 Discussion

Metabolic tumor volume and total lesion glycolysis derived from PET/CT scans provide a measure of active tumor regions and overall disease condition. There are no standardized processes for determining the regions for inclusion on MTV calculation, our study compared two processes to compute these estimates by manual (Reader M) and one semi-automated (Reader A) and its influence on the prognosis of treatment outcome.

There have been many studies that have shown utility in the use of PET imaging to estimate metabolic tumor volume (MTV) that has been shown relevance in many advanced diseases (43–45). Their few best practice guidelines for the clinical use of PET/CT, that provides recommendations for standardization of protocol to reduce variability, has been valuable to promote clinical usage (46, 47). Adoption to lymphoma disease has been qualitative as agreed consensus recommendation by the *Lugano classification scheme* (6, 48).

The metabolic tumor burden as a metric has been shown to be a marker of disease prognosis after treatment of axi-cel in DLBCL by our team and others (21, 24, 25, 29). In this study we compared estimates of MTV & TLG assessed by the readers, a clinical expert (Reader manual or M), and with imaging clinical experts (radiologists) (Reader auto or A). We compared these two estimates and showed that immaterial of the approaches, both metrics are prognostic of patient outcome to axi-cel treatment. In our prior study (25), using a training cohort of 48 patients and MTV (with a cutoff of 147.5 mL) was shown to be prognostic (p=0.005). Using the same cut off, we showed prognosis in an independent cohort of 48 patients (p=0.0003), which served to validate the approach. Others have shown a similar prognosis in a relatively smaller number of patients with p-value of 0.02 (49). There are many other studies that have tried to use clinical variables such as the standardized uptake value maximum (SUVmax) at different cutoffs to show patient prognosis after treatment (21). The current study outlines the role of lesion boundary correction on the MTV, TLG estimates using two approaches (M and A) and comparison of the influence of each on the clinical outcome, measured as over-all survival and progression free survival.

In this study, we find differences in MTV computations do exist due to: a) differences in detection, possibility due to limitation in using fixed threshold based reference, b) clinical interpretation between an inflammation and a lesion that may lead to accept, delete or alter a lesion, c) size based limitation included in detection algorithms, which allows to differentiate detected lesions with a metabolically active organ, both with a high SUV uptake (see **Figure 10**). Comparing MTV (Manual & Auto) there were four cases that switched prognosis (see **Table 4a**). It is interesting to note there were seven cases in total that switched prognosis groups comparing both MTV (Manual & Auto) and TLG (Manual & Auto), (see **Table 4**). Due to differences in weightage in computing metabolically active regions, prognostic decisions do affect comparing MTV and TLG. In our study there were 5 patients that switched prognostic groups in Manual estimates and 4 patients in Auto estimates (see **Table 4c, d**). While TLG between Manual and Auto showed a difference in prognosis for one patient (see **Table 4b**). We find these shifts are due to the patient’s metrics being close to the threshold point, adding to possible discrepancy in the decisions. In practice, patients close to cut points would need secondary validation or a follow up assessment to improve the strength of the prognosticator.

Some known issues in FDG-PET scan interpretations (37, 50) that are relevant to lymphoma have been listed (see Section 2.3). There are constraints in identifying metabolically active regions from normal physiology on the PET/CT scans (37). Some known issues include, altered tracer uptake in certain organs can be affected by medications, such as metformin related drugs that are known to increase in the colonic glycolysis, or increased bone marrow and splenic tracer uptake from hematopoietic stem cell factors given to the lymphoma patient population. Another example is focal increased FDG uptake in skeletal muscle related to physiologic muscular contraction by the patient in the PET scanner. Physiologic tracer uptake may thus occasionally lead to variability in interpretation between readers; despite the variabilities, there was a high concordance between the readers in our study.

Another critical factor affecting MTV measurements is the accurate discrimination of inflammation from lesions. Recognition of inflammatory processes involves identifying spatial and metabolic tracer patterns that do not match expected oncologic activity. Examples include tracer activity in anatomic regions that are distant from a locally advanced malignancy, tracer activity in anatomic regions not typical for a particular cancer’s pattern of spread (e.g. gall bladder update in prostate cancer or mesenteric fat necrosis in lymphoma), and treatment-related increased tracer uptake in tissues near tumor sites (i.e radiation therapy) (50) or distant from tumor sites. We would expect the concordance for such nodules to be low to moderate between the readers for these events.

It is evident that small changes in MTV seem to have a scaled effect in TLG metric, due to the multiplicative factor of SUVmax. We find, in the repeatability testing (Reader 1& 2), at certain ranges with small number of outliers (mid tertitle) influences the concordance coefficient (drops to 0.453 from 0.958 & 0.969 for smaller or larger tertiles).

Despite these potential reasons for discordance, the tumor metabolic volumes assessed by the two clinical readers (Reader A & M or Auto & Manual) showed high concordance and are prognostic to survival after treatment. We find the concordance correlation coefficient between readers dropped to 0.648 for lower tertiles and 0.882 for the highest tertiles, compared to 0.926 for the mid-range. It is evident that any changes made by the reader to patient studies with smaller tumor burden seem to impact the concordance metric. Alterations to larger region boundaries also makes a difference while assessing the MTV as the 41% on SUVmax significantly affects the boundary regions.

Conventional radiological assessment of solid tumors to date uses single dimensional lesions size measurements (RECIST, Response Evaluation Criteria in Solid Tumors) (51–53). It has been widely reported that there is a wide variability in tumor size estimations between the tumor measurements drawn by the clinical radiologists, which can be as much as 30% or greater, in some cases (17, 18). While use of PET/CT imaging in lymphoma provides both morphological and functional FDG-PET activity at the lesion level, availability of metabolic assessment at the lesion level allows for better convergence between the clinical readers. In DLBCL, the potentially large number of lesions found on body scans makes manual human interpretation exceptionally challenging. In our study, the patient cohort had an average of 12 lesions per patient, with a median of 6 and deviation of 16. Additional review time needed for detailed manual study interpretations makes the real time clinical implementation non practical. Methods for rapid and robust automated tumor assessments are desired, which is possible with the ongoing development of advanced imaging methods (54, 55).

It is nonetheless well understood that current detection methods have limitations, and human inputs are required for accurate disease assessment. Implementing a rule-based approach where a region of normal liver is used as a baseline measure provides for a more uniform disease burden assessment, as shown in this study. The study compares two clinical readers’ assessment of MTV/TLG estimates with slight differences in their approaches. The study clearly shows that at the population level any regional adjustments have minimal effect to outcome inference. The KM plot cut point for MTV estimates for Reader M using Manual method (86.75 mL) was higher compared to one with Reader A (63.55 mL), and comparable to previously reported clinical studies (24, 29), which in most cases followed manual approaches. The methods show a different cutoff range, which is evident on how lesion boundaries are adjusted to include perceived (manual or reader M) and following a rule-based cut point (auto or reader A). It is evident that following a procedure (manual or auto) one could translate the finding to derive prognosis at a patient level. It is cautioned that interchanging the MTV cutoff estimated by one method (manual or auto) may not be appropriate, due to subtle methodological differences. It is cautioned that, any inference or methodological preferences based on estimated prognosis threshold (Manual 86.76ml vs Auto 63.55ml) may not be appropriate. The study shows that smaller lesion level differences at the patient level will have minimal effect on the population level inferences for systemic disease with larger tumor burden such in DLBCL. Our study shows that using semi-automated lesion detection for MTV (or TLG) computation would provide statistically similar prognosis as a manual corrected lesions to estimate MTV, which we believe would allow development of automated methods and reducing clinical burden to estimate these metrics.

### 4.1 Study limitations

We acknowledge the limitations in our study, which include a relatively smaller patient sample size used for the research study. It has been understood that there are a very small number of institutions that offer advanced immunotherapy (axi-cell or similar), making the patient samples scares. We made our best effort to assemble a sample size that would allow us to obtain statistically meaningful observations. We understand that the study findings need independent secondary validation.

## 5 Conclusion

The study systematically compares two reader approaches who independently estimated MTV and TLG assessment. The use of a semi-automated approach to lesion identification allows greater clinical adoption due to ease in computation and lessens the need for manual contouring. We conclude that lesion boundary alterations have minimal effects in the population level prognosis of outcome with both methods with comparable statistical significance. Our study supports development of automated methods for determination of MTV to improve clinical throughput and reduce reader biases.

## Data availability statement

The original contributions presented in the study are included in the article/**Supplementary Material**. Further inquiries can be directed to the corresponding authors.

## Ethics statement

The study protocol was approved by the Institutional Review Board at the University of South Florida/Moffitt Cancer Center.

## Author contributions

JC, ED, FL, YB: Hypothesis, methods direction. FL, ED, MJ, GK: Clinical data curation, clinical care. HL, JQ, JC, ED: Lesions identification and marking. YB, ZT: Results inference, implementation of methods. YB, FL, JC, ED Manuscript writing and editing. JC, ED, HL, JQ, ZT, GK, MJ, FL, YB: Manuscript proof-read and approval. All authors contributed to the article and approved the submitted version.
