# Supplementary material for: Repeatability of metabolic tumor burden and lesion glycolysis between clinical readers
Source: Front Immunol. 2023 Feb 15;14:994520. doi: 10.3389/fimmu.2023.994520 (PMC9975754; doi:10.3389/fimmu.2023.994520)
Supplement: Supplementary file 1 [file Table_1.docx]

**SUPPLEMENTAL DATA**

**Suppl. Table 1**. Cox regression model to assess role of MTV to overall survival (1 year). The estimate variable a) tertiles on MTV and b) tertiles on number of lesion to assess hazard risk..

1. MTV tertiles

| **Reader M** | | | | | |
| --- | --- | --- | --- | --- | --- |
|  | **Variable** | | **Hazard Ratio** | | **P-value** |
|  | MTV (Reader M) | | 1.00147 | | 0.00149 |
|  | Bridge Therapy | | 1.0286 | | 0.937 |
| Tertile on MTV #1 [1.7, 41.3] | | | | | |
|  | MTV | | 1.05 | | 0.0326 |
|  | Bridge Therapy | | 0.687 | | 0.559 |
|  | Lesion count | | 1.0332 | | 0.4337 |
| Tertile#2 [41.3, 259.8] | | | | | |
|  | MTV | | 1.05 | | 0.0326 |
|  | Bridge Therapy | | 0.687 | | 0.559 |
|  | Lesion count | | 1.033 | | 0.423 |
| Tertile#3 [259.8, 1276.1] | | | | | |
| MTV | | 0.999 | | 0.972 | |
| Bridge Therapy | | 1.252 | | 0.668 | |
| Lesion count | | 1.0006 | | 0.490 | |

| **Reader A** | | | | | |
| --- | --- | --- | --- | --- | --- |
|  | **Variable** | | **Hazard Ratio** | | **P-value** |
|  | MTV (Reader A) | | 1.00104 | | 0.018 |
|  | Bridge | | 1.1496 | | 0.692 |
| Tertile#1 [1.7, 41.3] | | | | | |
|  | MTV | | 1.0034 | | 0.876 |
|  | Bridge Therapy | | 2.256 | | 0.339 |
|  | Lesion count | | 1.041 | | 0.651 |
| Tertile#2 [41.3, 259.8] | | | | | |
|  | MTV | | 1.0085 | | 0.066 |
|  | Bridge Therapy | | 0.695 | | 0.567 |
|  | Lesion count | | 1.0494 | | 0.225 |
| Tertile#3 [259.8, 1276.1] | | | | | |
| MTV | | 0.9992 | | 0.351 | |
| Bridge Therapy | | 1.4748 | | 0.445 | |
| Lesion count | | 1.0055 | | 0.545 | |

1. Number of lesions

| **Reader M** | | | | | |
| --- | --- | --- | --- | --- | --- |
|  | **Variable** | | **Harzard Ratio** | | **P-value** |
|  | MTV (Reader M) | | 1.000085 | | 0.00857 |
|  |  | |  | |  |
| Tertile on MTV #1 [1, 4) | | | | | |
|  | MTV | | 1.005 | | 0.025 |
|  | Bridge Therapy | | 4.33 | | 0.077 |
|  | Lesion count | | 0.826 | | 0.783 |
| Tertile#2 [4, 11) | | | | | |
|  | MTV | | 1.0008 | | 0.338 |
|  | Bridge Therapy | | 0.811 | | 0.760 |
|  | Lesion count | | 1.294 | | 0.110 |
| Tertile#3 [11, 91] | | | | | |
| MTV | | 1.0018 | | 0.035 | |
| Bridge Therapy | | 0.876 | | 0.806 | |
| Lesion count | | 0.996 | | 0.794 | |

| **Reader A** | | | | | |
| --- | --- | --- | --- | --- | --- |
|  | Variable | | Harzard Ratio | | P-value |
|  | MTV (Reader A) | | 1.0011 | | 0.00656 |
|  |  | |  | |  |
|  | ***Multivariable*** | |  | |  |
|  | MTV | | 1.001044 | | 0.018 |
|  | Bridge Therapy | | 1.1497 | | 0.692 |
|  | | | | | |
| Tertile#1 [1, 4) | | | | | |
|  | MTV | | 1.0048 | | 0.0285 |
|  | Bridge Therapy | | 4.2005 | | 0.0813 |
|  | Lesion count | | 0.816 | | 0.769 |
| Tertile#2 [4, 11) | | | | | |
|  | MTV | | 1.00054 | | 0.4489 |
|  | Bridge Therapy | | 0.8557 | | 0.8198 |
|  | Lesion count | | 1.3059 | | 0.0945 |
| Tertile#3 [11, 91] | | | | | |
| MTV | | 1.001 | | 0.134 | |
| Bridge Therapy | | 1.1159 | | 0.825 | |
| Lesion count | | 1.00347 | | 0.772 | |

**Suppl. Table 2**. Cox regression model to assess role of TLG to overall survival (1 year). The estimate variable a) tertiles on TLG and b) tertiles on number of lesion to assess hazard risk.

1. TLG tertile

| **Reader M** | | | | | |
| --- | --- | --- | --- | --- | --- |
|  | **Variable** | | **Harzard Ratio** | | **P-value** |
|  | TLG (Reader M) | | 1.00008514 | | 0.00857 |
|  | ***Multivariable*** | |  | |  |
|  | TLG | | 1.0000792 | | 0.0249 |
|  | Bridge Therapy | | 1.16069 | | 0.6735 |
| Tertile on TLG #1 [4.35, 319] | | | | | |
|  | TLG | | 1.006 | | 0.129 |
|  | Bridge Therapy | | 1.420 | | 0.647 |
|  | Lesion count | | 0.966 | | 0.749 |
| Tertile#2 [41.3, 259.8] | | | | | |
|  | TLG | | 1.0006 | | 0.077 |
|  | Bridge Therapy | | 1.203 | | 0.755 |
|  | Lesion count | | 1.037 | | 0.198 |
| Tertile#3 [259.8, 1276.1] | | | | | |
| TLG | | 1.0000086 | | 0.893 | |
| Bridge Therapy | | 0.8695 | | 0.793 | |
| Lesion count | | 1.00811 | | 0.364 | |

| **Reader A** | | | | | |
| --- | --- | --- | --- | --- | --- |
|  | Variable | | Harzard Ratio | | P-value |
|  | TLG (Reader A) | | 1.0000734 | | 0.0313 |
|  | ***Multivariable*** | |  | |  |
|  | TLG | | 1.0000657 | | 0.0703 |
|  | Bridge therapy | | 1.23354 | | 0.5443 |
| Tertile#1 [1.7, 41.3] | | | | | |
|  | TLG | | 1.0083 | | 0.07 |
|  | Bridge Therapy | | 1.595 | | 0.551 |
|  | Lesion count | | 0.981 | | 0.866 |
| Tertile#2 [41.3, 259.8] | | | | | |
|  | TLG | | 0.999 | | 0.726 |
|  | Bridge Therapy | | 1.261 | | 0.691 |
|  | Lesion count | | 1.039 | | 0.171 |
| Tertile#3 [259.8, 1276.1] | | | | | |
| TLG | | 1.000013 | | 0.997 | |
| Bridge Therapy | | 0.883 | | 0.816 | |
| Lesion count | | 1.0081 | | 0.365 | |

1. Number of lesions

| **Reader M** | | | | | |
| --- | --- | --- | --- | --- | --- |
|  | **Variable** | | **Harzard Ratio** | | **P-value** |
|  | **TLG (Reader M)** | | 1.000085 | | 0.00857 |
|  |  | |  | |  |
| Tertile on TLG #1 [1, 4) | | | | | |
|  | TLG | | 1.0002 | | 0.124 |
|  | Bridge Therapy | | 3.609 | | 0.106 |
|  | Lesion count | | 1.027 | | 0.966 |
| Tertile#2 [4, 11) | | | | | |
|  | TLG | | 1.0005 | | 0.392 |
|  | Bridge Therapy | | 0.834 | | 0.760 |
|  | Lesion count | | 1.308 | | 0.097 |
| Tertile#3 [11, 91] | | | | | |
| TLG | | 1.0018 | | 0.047 | |
| Bridge Therapy | | 0.753 | | 0.635 | |
| Lesion count | | 0.995 | | 0.685 | |

| **Reader A** | | | | | |
| --- | --- | --- | --- | --- | --- |
|  | Variable | | Harzard Ratio | | P-value |
|  | TLG (Reader A) | | 1.0000734 | | 0.0313 |
|  |  | |  | |  |
| Tertile#1 [1, 4) | | | | | |
|  | TLG | | 1.0003 | | 0.071 |
|  | Bridge Therapy | | 4.299 | | 0.086 |
|  | Lesion count | | 0.915 | | 0.892 |
| Tertile#2 [4, 11) | | | | | |
|  | TLG | | 1.00005 | | 0.419 |
|  | Bridge Therapy | | 0.843 | | 0.805 |
|  | Lesion count | | 1.309 | | 0.095 |
| Tertile#3 [11, 91] | | | | | |
| TLG | | 1.00007 | | 0.31 | |
| Bridge Therapy | | 1.182 | | 0.735 | |
| Lesion count | | 1.0048 | | 0.682 | |
